# 

**Published:** 1907-11-30

**Authors:** 


### CONTENTS.

| | PAGE |
|---|---|
| **Medical Education and the London University** | 225 |
| **Relapses in Diphtheria** | 226 |
| **Annotations—** | |
| The Diagnosis of Rheumatic Fever—London's Water Supply—Aberdeen University Club, London | 227 |
| **Medical Opinion and Movement** | 228 |
| **Hospital Clinics—** | |
| Some Medico-Legal Reminiscences. By Arthur P. Luff, M.D., B.Sc., F.R.C.P. (Lond.) | 229 |
| Abdominal Tuberculosis in Childhood. By William P. S. Branson, M.D., M.R.C.P. | 231 |
| **Clinical Points—** | |
| Albuminuria in Phthisis | 233 |
| Spontaneous Fragmentation of Urinary Calculi | 234 |
| **Points in Surgery—** | |
| The Treatment of Fractures from a Common-sense Point of View | 235 |
| **Anatomical Points—** | |
| The Lymphatic Glands of the Face | 236 |
| **Diseases of Children—** | |
| Hyperpyrexia | 237 |
| **Gynæcology—** | |
| Maternal Nursing | 238 |
| **Ophthalmology—** | |
| Injuries to the Eye | 239 |
| **Otology—** | |
| Concerning Operations for Otorrhœa | 240 |
| **Public Health and Hygiene—** | |
| The Cold Summer and the Death Rate | 241 |
| Diagram of the Weekly Death Rate in 1907 | 242 |
| **Practical Notes on Diagnosis and Treatment** | 242 |
| **Resident Medical Officers' Department—** | |
| Picric Acid Poisoning | 243 |
| **Therapeutics—** | |
| Prescriptions and Dispensing | 244 |
| **Hospital Administration—** | |
| The Sick and Infirm | 245 |
| The Bristol Royal Infirmary | 246 |
| The Graduates' Medical Schools | 247 |
| Medical Education in London | 247 |
| Practical Points | 248 |
| News and Coming Events | 248 |
| **Nursing Administration—** | |
| The Registration of Nursing Homes | 249 |
| **Editor's Letter-Box—** | |
| The Cinematograph in Medicine | 250 |
| Nurse-Matrons', Sisters', and Dispensers' Certificates | 250 |

